# Impact of Jaw-Sucking Movements on Postural Muscles Tension in Young Adults

**DOI:** 10.3390/jcm15041464

**Published:** 2026-02-13

**Authors:** Agnieszka Ptak, Małgorzata Stefańska

**Affiliations:** Faculty of Physiotherapy, Wroclaw University of Health and Sport Science, al. Paderewskiego 35, 51-612 Wrocław, Poland; malgorzata.stefanska@awf.wroc.pl

**Keywords:** jaw movement, tongue position, muscle function, electromyography (EMG), myotonometry

## Abstract

**Background:** The objective of this study was to assess the tension of selected postural muscles during the jaw-sucking movement in four body positions (standing position, all-fours position, lying on front, lying on the side). **Material and Methods:** The research involved 30 young adults with an average age of 22.6 ± 0.72 years. Suprahyoid, trapezius, gluteus maximus, and gastrocnemius muscles were assessed in all study participants in the standing, kneeling, and belly lying positions (prone position). Measurements were taken twice for each position: once without jaw activity and once with jaw movements simulating sucking. Muscle function was determined by measuring muscle tension using surface electromyography (sEMG). **Results**: Engaging jaw movements in the prone position resulted in significantly increased tension in the gastrocnemius muscle. In the all-fours position, there was a notable rise in tension in both the gastrocnemius and gluteus maximus muscles. When standing, significantly higher tension was observed in the trapezius and gluteus maximus muscles. In contrast, the side-lying position exhibited no significant changes in muscle tension. **Conclusions:** The study’s findings suggest that activating jaw function may affect the tone of the gastrocnemius muscle in both prone and quadrupedal positions. In contrast, there were no clear or statistically significant changes observed in the tone of trapezius muscles in either position, while, for the tension of the gluteus medius muscle, variability was shown only in the all-fours position.

## 1. Introduction

Research shows that jaw clenching activates neural pathways connecting the trigeminal system, which includes the jaw muscles, with the reticular and spinal networks. Both chewing and jaw clenching reliably activate the bilateral primary motor cortex, supplementary motor area, thalamus, and cerebellum. However, chewing, which is a dynamic movement, shows greater activation in the left primary somatosensory cortex compared to jaw clenching, which is a static force. This leads to increased excitability of spinal motor neurons that control lower limb muscles [1].

The resting position of the tongue and jaw, particularly during clenching, is correlated with reflexive muscle activity that activates the muscles of the lower limbs, especially the gastrocnemius and soleus muscles [2,3]. Research indicates that tongue positioning can influence postural control mechanisms. Positioning the tongue against the upper incisors may improve postural stability while standing upright on an unstable surface [3].

This mechanism involves both intercortical connections, which activate multiple motor areas in the brain, and heightened spinal excitability, leading to increased activity of α-motor neurons and muscle spindles. This relationship is most apparent in healthy adults [2]. Research indicates that the jaw and neck regions are interconnected anatomically, biomechanically, and neurologically. Studies have shown that voluntary clenching can enhance muscle strength and improve performance in various motor tasks. Additionally, information from the neck’s sensory-motor system plays a crucial role in maintaining posture and leading to greater posture stability [4]. Therefore, it is reasonable to conclude that activating the jaw’s sensory-motor system may influence posture as well [4]. Co-activation amplification (CAP) is thought to increase muscle strength by contracting muscles distant from the primary mover. Concurrent activation potentiation (CAP) was first defined as the phenomenon that leads to an acute increase in muscular and athletic performance, achieved by simultaneously activating muscles not directly involved in the activity [5]. This phenomenon has been described as remote voluntary contractions (RVCs) [6]. The simultaneous activation of muscles not directly involved in the primary movement is referred to as remote voluntary contraction (RVC). RVC strategies vary from single RVC, which involves the activation of only one muscle group, to combined RVCs, where multiple muscle groups are activated at the same time [7]. The research highlights a significant relationship between the intensity and quantity of concomitant reflexive voluntary contractions (RVCs) and enhanced performance in strength tasks. Specifically, engaging in multiple RVCs, such as hand gripping, jaw clenching, and performing the Valsalva maneuver, during maximal isometric leg extension tasks, has been shown to produce greater overall strength outputs compared to executing each RVC individually. This suggests that the combined effects of these muscle contractions can synergistically elevate strength levels, indicating a remarkable interplay between these voluntary movements and overall physical performance [6]. At this moment, these findings are used in combat sports and athletes as well, leading to increased force muscle and better postural stability [5,7,8].

Human motor development begins in the forward lying position—from the moment of the first crawling movement made towards the mother’s breast. During primitive crawling, muscle chains are activated, and then used in the process of locomotion [9,10,11]. Developmental kinesiology, which describes the interaction between the nervous system, the muscular system, and the osteoarticular system, has been described in many studies and is used, among other things, in sports training [12,13,14,15]. Postural control allows you to change the position of the body in space, achieving an upright posture by overcoming the force of gravity. Posture stabilization involves movement of the limbs and head independently of the trunk [16,17]. During crawling, muscle chains are activated, allowing the lower limbs to move around the mother’s body. These movements are essential for walking in adulthood. During the walking movement, the gluteus maximus and gastrocnemius muscles, among others, are involved in the take-off phase [18,19].

The observed reflex mechanisms are described in the literature [10,20]. Their identification was based on observations. However, there is a lack of assessment of the ongoing process using objective research tools.

This study aims to objectively evaluate the tension of specific postural muscles during jaw-sucking movements across various body positions. The measurements were performed in a group of young healthy adults whose sucking was forced by using a pacifier. The study aimed to functionally assess selected postural muscles during the sucking process.

## 2. Material and Method

The study was designed as an experimental, repeated-measures investigation involving a single group of participants. Each participant underwent all experimental conditions, allowing for within-subject comparisons across repeated, single-session measurements.

The study group comprises young, healthy adults, specifically university students, to facilitate a uniform sample that is consistent across parameters such as physical activity, knowledge level, and health status. This selection criterion establishes the participant age range from 20 to 24 years, ensuring that all individuals are in optimal health. Inclusion criteria for the study included: being between the ages of 20 and 24 years, having no active or chronic diseases affecting the musculoskeletal system or neurological disease, and providing consent to participate. Exclusion criteria included any limitations in gross motor function, disorders of the orofacial area, or abnormal muscle tone resulting from disease processes. Additionally, individuals with a body mass index (BMI) below 18.5 (underweight) or above 25 (overweight or obese) were excluded. Those with active or chronic diseases affecting the trunk and limbs were also excluded.

The sample size was calculated using the G*Power 3.1 program, aiming for a minimum test power of 0.8 and an effect size of 0.5. As a result, the minimum required group size for two repeated measurements (using the Wilcoxon test or T test for dependent measures) was determined to be 27 or 28 participants. An average dropout rate of 10% was assumed.

The research involved 30 young adults (21 women and 9 men), physiotherapy students of the University of Health and Sport Science in Wrocław. Participants in the study were recruited randomly from those who entered the study. Detailed characteristics of the group are presented in Table 1.

### 2.1. Research Design

The study group consisted of young, healthy adults, specifically university students aged 20 to 24 years.

Each person joining the project was familiarized with the purpose and procedure of the study and provided written consent to participate. Initially, each participant completed a short questionnaire containing sociodemographic characteristics and information enabling qualification for the study group (inclusion and exclusion criteria). In preparation for the EMG study, skin was prepared and disposable Ag/AgCl electrodes were applied along the muscle fibers, at the widest point of the muscle belly: the suprahyoid muscle, trapezius muscle, gluteus maximus muscle, and gastrocnemius muscle. Each participant received a disposable pacifier. The measurements were preceded by a detailed description of the study, including the use of the pacifier and descriptions of the individual measurement positions (standing, all-fours, and lying on the front and side). Each participant, under the researcher’s supervision, performed a test sucking motion for approximately 30 s, ensuring maximum force was achieved under the measurement conditions. The first measurement was recorded in the standing position without sucking simulation, followed by the sucking simulation. The standing position required the body to assume a relaxed position without active postural correction. Next, the measurement was performed in the all-fours position without sucking simulation, and then with sucking simulation. The all-fours position involved supporting the body on both hands and knees while keeping the spine in a neutral alignment. Subsequent measurements were taken in two different positions: first in the front-lying (prone) position, where the participant lay face down with the head in a neutral position, and then in the side-lying (lateral) position, where the body was aligned laterally with the head supported to maintain proper alignment. Each EMG recording was initiated when muscle tension stabilized after the position change. A 5 s recording of muscle activity was recorded and then analyzed.

All participants were informed about the aims and procedures of the study and provided voluntary written informed consent before taking part. They were also made aware of their right to withdraw from the study at any time without any consequences. The research adhered to the principles of the Declaration of Helsinki and received approval from the Senate Commission for the Ethics of Scientific Research at the University of Health and Sport Sciences in Wroclaw. Written consent was obtained for participation, data analysis, and the anonymous publication of the results.

### 2.2. Measurement

Muscle tension (µV) was assessed in the suprahyoid, trapezius, gluteus maximus, and gastrocnemius (lateral part) muscles for all study participants in standing, kneeling, and prone-lying positions. All measurements were taken on the left side of the body. Participants were instructed to engage in four distinct postural positions: standing, quadruped, prone (belly lying), and lateral (side-lying), while utilizing a sucking pacifier. To simulate sucking, standard-shaped baby pacifiers made of silicone in the largest size available (18 m+) were used. During both activities, the measurement of parameters of the muscle was taken. Muscle tension recorded in a relaxed standing position was taken as the reference value (100%).

**Figure 1:**
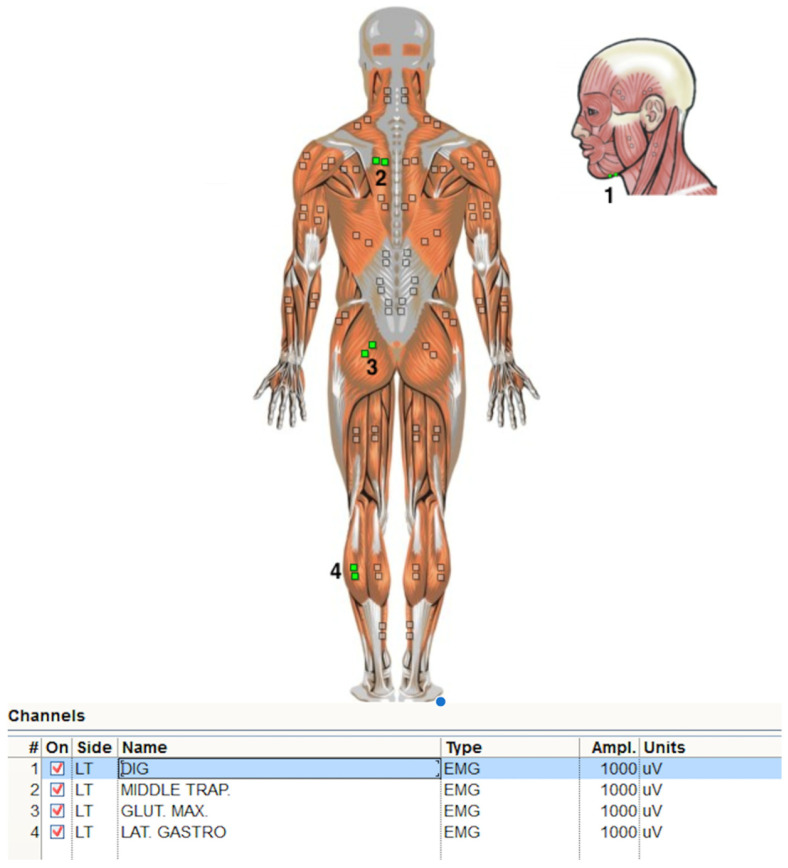
Electrode placement 1—suprahyoid muscles, 2—middle trapezius, 3—gluteus maximus, 4—lateral gastrocnemius by Noraxon Inc.

Muscle function was determined by measuring muscle tension using surface electromyography (sEMG). Voltage measurements were performed using a 4-channel wireless electromyograph Myo Trace 400 by Noraxon Inc. (Scottsdale, AZ, USA). The research procedure was carried out following the principles presented in the SENIAM project (24). Disposable pediatric gel electrodes (Ag/AgCl) with a conduction diameter of 1 cm were glued along the muscle fibers in the widest part of the belly muscle so that the centers of the electrodes were approximately 3 cm apart on the left side of the body. Surface EMG electrodes were placed according to the Noraxon EMG manual and SENIAM guidelines, with bipolar Ag/AgCl electrodes aligned parallel to muscle fibers and an interelectrode distance of ~20 mm. Electrodes for the suprahyoid muscles were positioned submentally over the muscle belly between the mandibular symphysis and the hyoid bone, avoiding the midline. The middle trapezius electrodes were placed midway between the spine and the medial border of the scapula at T3. The gluteus maximus electrodes were positioned over the central muscle belly between the sacrum and the greater trochanter. The lateral gastrocnemius electrodes were placed on the most prominent part of the lateral muscle belly, approximately one third of the distance from the fibular head to the calcaneus (Figure 1). The reference electrode was glued to the acromion of the scapula. To create good impedance conditions, the skin was shaved and degreased with a 70% alcohol solution before electrode application. Measurements were performed in three positions: standing, quadruped, prone (belly lying), and lateral (side-lying). In each position, the EMG signal was recorded twice: while standing/lying freely and during sucking forced by using a baby pacifier. The subjects were instructed to exert maximal suction effort during the task. The signal was recorded every 5 s. Signal recording and processing were performed using the software MR-XP MT400 4CH Master Edition 1.08.38. The EMG signal spectrum recording was smoothed by using the RMS algorithm in a time window of 5 ms and a band-pass filter with cut-off frequencies of 10–500 Hz. The measurement was performed with a sampling frequency of 1500 Hz.

The average and maximum voltage amplitude values (µV) were determined for each measurement. Amplitude values obtained with the all-fours position in lying positions (with and without sucking) were normalized to values recorded in a standing position without simultaneous sucking. Normalization required calculating what percentage of the voltage amplitude obtained in a standing position without simultaneous sucking was obtained while lying on the side or front and the all-fours position. The values obtained in the standing position without sucking simulation were treated as the reference value (100%) (Tables S1 and S2).

### 2.3. Data Collection

Data were collected from April to May 2024. All measurements were conducted by the authors of the article, who are researchers and physiotherapists. The data were recorded in a coded manner and are stored in the central research laboratory. The experimental protocol received approval from the Senate Committee for Scientific Research Ethics. The research project was carried out in the Central Laboratory University of Health and Sport Science in Wrocław with the consent of the Senate Committee for Scientific Research Ethics No. 4/2024.

### 2.4. Data Analysis

The distribution of all analyzed variables was checked using the Shapiro–Wilk test. Most variables did not have a distribution close to normal. The *p*-values of the Shapiro–Wilk test were less than 0.05. Descriptive statistics were calculated. The median was used as a measure of central tendency, and the interquartile range (IQR) was used as a measure of dispersion. The significance of differences between variables was determined using the Wilcoxon pairwise order test. The effect size for the Wilcoxon was tested using the r coefficient was calculated according to the formula r = $Z/\sqrt{N}$ (where *Z*—test value, *N*—number of pairs) [21]. The level of significance was set to *p* < 0.05. The analysis was performed in Statistica 13.1.

## 3. Results

The activity of selected muscle groups in four positions of the body was analyzed twice in the measurement, without sucking (without) and during sucking (sucking). The obtained sEMG signal values (µV) were normalized to the values recorded in a standing position without sucking. This value was treated as 100%. All other measurements were presented as a percentage of the reference value. It was observed that, in the standing position, during the sucking simulation, significantly higher tension was observed not only in the suprahyoid muscle but also in the trapezius and gluteus maximus muscles compared to the values measured without sucking. No significant differences were observed only for the triceps calf muscle (Table 2).

Measurements conducted in the all-fours position and in the front-lying position showed a significant increase in the triceps calf muscle tension during sucking simulation compared to measurements without jaw activity (Table 3 and Table 4).

Comparing the values obtained in the lateral recumbency position with and without sucking simulation, no significant changes in postural muscle tone were observed after jaw activation. The exception was a significant increase in mean trapezius muscle tone (Table 5).

## 4. Discussion

This study aimed to investigate the potential of jaw-sucking movements to enhance co-activation amplification (CAP) and its effects on lower limb activity in positions other than standing. The study showed that, during the sucking simulation, the activity of certain postural muscles increased. Depending on the test position, changes in mean and maximum muscle tone were observed in the trapezius and gluteus maximus muscles in the standing position, the gastrocnemius muscles in the quadruped position, and in a prone/belly lying position. Additionally, a significant increase in mean muscle tone was observed in the gluteus maximus muscles in the quadruped position and in the trapezius muscles in a side-lying position. In most statistically significant analyses, the demonstrated relationships were confirmed by high effect sizes (>0.05). Moderate effect sizes were obtained only in the quadruped position for moderate gluteus medius contraction and in the front-lying position for maximum triceps contraction. People clench their teeth to activate facial, neck and abdominal muscles when they need to generate heavy muscle force against large resistance, like lifting heavy objects. These are termed as remote voluntary contraction (RVC) [22]. An analysis of existing research was conducted to examine muscle activity during forceful activities, specifically considering the role of jaw clenching as a co-activation mechanism in young adults. This investigation aimed to elucidate the relationship between jaw clenching, hand grip force, and countermovement jump performance [5]. Co-activation of muscles has also been historically observed through the Jendrasik maneuver in individuals with neurological impairments [3].

Authors conclude that the co-activation of jaw muscles influences muscle force and leads to better posture stability [23]. Those findings were applicable in combat sports and the rugby leagues [1]. Research has predominantly focused on activities conducted in either a standing position or during movement, such as the counterjump, which was typically performed while standing [3,4,7] or sitting [8]. Additionally, authors consider tongue position to be a crucial factor in the co-activation process [3]. Tongue placement affects postural control; resting it against the upper incisors may improve stability when standing on unstable surfaces [3]. This type of movement engages all muscle groups. The described movement sequences can be seen during breastfeeding, crawling, and tummy time [24]. Movement of the jaw and tongue position is mainly discussed according to the latching process in newborn babies and tummy time at an early stage [10,18,25,26,27,28]. Both activities performed at the belly position are strongly correlated with jaw-sucking movements. They actively influence the entire body, from the head to the toes [10], and allow the use of two components of movement in the temporomandibular joint hinge movement and sliding movement connected with tongue movement, recognized as a jaw-sucking-like movement [10,29,30,31]. Co-activation of the lower limb is observed during latching when the newborn places itself on the mother’s breast [32].

Our research shows a change in the average and maximum tension of the gastrocnemius muscle during jaw-sucking movements, similar to the lower limb activity during the push-off phase of walking. This aligns with muscle reactivity seen in developmental kinesiology. We recommend using the prone and quadruped positions to promote proper motor development [24,33]. Similar conclusions are suggested in works by authors examining the breastfeeding process using a belly-to-belly position [10] and tummy time position [33].

The anatomical and functional relationship between the mandible and the gastrocnemius muscle is elucidated through the concept of myofascial chains. While these two regions are not directly connected anatomically, a functional interdependence is evident due to the myofascial connections that bridge them [34]. The main anatomical considerations encompass the deep fascia of the gastrocnemius, which exhibits a proximal connection to the hamstrings and pelvis via the popliteal region and the sacrotuberous ligament. This connection constitutes a significant component of the “superficial back line” as delineated in myofascial chain models [35]. Myofascial chains show that movement or tension in one area, like the jaw, can affect muscle tone elsewhere, such as the lower limb, due to fascia interconnection. Research has proven that movement in one region, like the pelvis, can cause measurable displacement in the gastrocnemius fascia, highlighting force transmission across the body [34,35].

Muscle activity analysis during jaw-sucking movements in the prone position using objective research tools supports previous findings from partial models and subjective studies. Body position significantly influences muscle activation patterns and the relationship between sucking activity and postural muscle engagement. Variations in core and trunk muscle recruitment are noted in positions like quadruped, prone, and side-lying, impacting both postural stability and orofacial muscle function [36]. In the quadruped position, there is considerable bilateral activation of the lumbar multifidus, erector spinae, and oblique muscles, which elevates the demand for trunk stabilization. This increased muscular engagement is critical for maintaining postural integrity and optimizing functional movement patterns [36]. In the prone position, characterized by lying on the belly, there is a pronounced activation of the rectus abdominis, along with the internal and external obliques. Furthermore, the engagement of the latissimus dorsi, in conjunction with lower limb movement, facilitates an augmentation of both core and gluteal muscle activation. This interaction underscores the importance of body orientation in optimizing muscular engagement during physical activities [37]. Adopting a side-lying position leads to a reduction in overall trunk muscle activation, particularly on the supported side. This positioning is associated with decreased fatigue of the erector spinae muscles, potentially facilitating a state of relaxation [38]. In our own research, we observed that, during the active sucking phase, there was an increase in muscle tension in areas not directly related to the sucking process. Achieving postural stabilization while in the front-lying or all-fours positions activates both postural and orofacial muscles. This activation, facilitated by musculoskeletal chains, also engages the muscles of the lower limbs [1,3,36,39].

## 5. Recommendation

The robust movements of the jaw, observed in both quadruped and prone positions, suggest a connection to the co-activation of postural muscles. This relationship may influence the activation and enhancement of human motor skills. The findings indicate that this dynamic engagement of kinematic chains could significantly contribute to the development of muscular strength and motor coordination. Moreover, this connection may warrant further exploration in training and therapeutic contexts across different age groups, including both adults and children.

## 6. Limitation

The study concentrated on specific muscle groups and included only young adult participants. Furthermore, suction strength was not measured objectively; participants were simply instructed to suck as hard as they could. These limitations should be considered when interpreting the findings, as they may restrict the applicability of the results to different age groups or populations with diverse functional characteristics.

## 7. Conclusions

The study’s findings indicate that activating jaw function may influence the tone of the gastrocnemius muscle when in both prone and quadrupedal positions. In contrast, there were no clear or statistically significant changes observed in the tone of trapezius muscles in either position, while, for the tension of the gluteus medius muscle, variability was shown only in the all-fours position.

## Figures and Tables

**Table 1:** Characteristics of the study group.

	Mean	SD
Age (years)	22.60	0.72
Body height (cm)	171.40	8.89
Body weight (kg)	65.65	10.37
BMI (kg/m^2^)	22.23	2.16
Physical activity (hours/week)	6.10	3.80

BMI—body mass index, SD—standard deviation.

**Table 2:** Mean and maximum muscle tension in the standing position with simultaneous sucking, normalized to the standing position without sucking.

Standing Position		With Sucking Simulation		Wilcoxon’s *p*	Effect Size
Muscle	Tension	Median	IQR		r
m. suprahyoid	max (%)	2375.72	1877.87	0.0001 *	0.87
	mean (%)	1708.99	1239.56	0.0001 *	0.87
m. trapezius	max (%)	111.87	71.16	0.0220 *	0.44
	mean (%)	114.81	90.69	0.0312 *	0.42
m. gluteus maximus	max (%)	119.45	31.05	0.0014 *	0.62
	mean (%)	113.75	39.12	0.0094 *	0.50
m. gastrocnemius	max (%)	85.90	77.16	0.8817	0.03
	mean (%)	69.33	67.38	0.2173	0.24

IQR—interquartile range, m.—muscle, * *p* < 0.05.

**Table 3:** Average and maximum muscle tension in the quadruped position, normalized to the standing position without sucking.

Quadruped Position		Without Sucking		With a Sucking Simulation		Wilcoxon’s *p*	Effect Size
Muscle	Tension	Median	IQR	Median	IQR		r
m. suprahyoid	max (%)	179.94	149.80	2770.46	1691.80	<0.0001 *	0.87
	mean (%)	170.96	130.25	2124.94	1051.73	<0.0001 *	0.87
m. trapezius	max (%)	87.09	93.00	103.39	77.15	0.6964	0.08
	mean (%)	110.69	94.33	104.28	65.04	0.4926	0.13
m. gluteus maximus	max (%)	118.05	58.80	133.62	99.60	0.0546	0.37
	mean (%)	119.73	45.98	117.33	59.04	0.0409 *	0.40
m. gastrocnemius	max (%)	21.43	25.22	24.68	34.95	0.0042 *	0.60
	mean (%)	24.32	26.55	36.21	53.77	0.0003 *	0.76

IQR—interquartile range, m.—muscle, * *p* < 0.05.

**Table 4:** Average and maximum muscle tension in a prone position/belly lying position normalized to the standing position without sucking.

Prone Position		Without Sucking		With a Sucking Simulation		Wilcoxon’s *p*	Effect Size
Muscle	Tension	Median	IQR	Median	IQR		r
m. suprahyoid	max (%)	148.57	185.97	2200.65	918.80	<0.0001 *	0.88
	mean (%)	181.53	199.29	1894.13	995.19	<0.0001 *	0.87
m. trapezius	max (%)	73.34	106.47	84.85	110.98	0.3533	0.18
	mean (%)	90.70	114.36	83.27	71.38	0.4926	0.13
m. gluteus maximus	max (%)	101.82	53.79	99.85	60.24	0.9893	0.00
	mean (%)	96.98	30.78	103.84	38.17	0.4758	0.14
m. gastrocnemius	max (%)	21.31	25.39	21.39	23.97	0.0385 *	0.40
	mean (%)	21.71	25.27	25.28	21.86	0.0051 *	0.55

IQR—interquartile range, m.—muscle, * *p* < 0.05.

**Table 5:** Average and maximum muscle tension in a side-lying position, normalized to the standing position without sucking.

In a Side-Lying Position		Without Sucking		With a Sucking Simulation		Wilcoxon’s *p*	Effect Size
Muscle	Tension	Median	IQR	Median	IQR		r
m. suprahyoid	max (%)	169.86	142.13	2119.35	2171.92	<0.0001 *	0.87
	mean (%)	152.85	84.81	1745.81	1376.95	<0.0001 *	0.87
m. trapezius	max (%)	72.97	74.60	112.84	99.26	0.1300	0.31
	mean (%)	68.45	49.20	101.26	96.69	0.0074 *	0.57
m. gluteus maximus	max (%)	87.10	47.77	92.09	39.77	0.4564	0.14
	mean (%)	94.32	27.00	93.29	28.05	0.2588	0.22
m. gastrocnemius	max (%)	16.33	25.90	16.87	26.92	0.8314	0.04
	mean (%)	25.38	38.33	25.45	37.99	0.1374	0.30

IQR—interquartile range, m.—muscle, * *p* < 0.05.

## Data Availability

The data that support the findings of this study are not openly available due to reasons of sensitivity and are available from the corresponding author upon reasonable request. Data are located in controlled-access data storage.

## Supplementary Materials

The following supporting information can be downloaded at: https://www.mdpi.com/article/10.3390/jcm15041464/s1, Table S1. Average and maximum muscle tension during sucking simulation depending on body position, normalized to the standing position; Table S2. Average and maximum muscle tension in measurement without sucking simulation, depending on body position, normalized to the standing position.
