# Supplementary material for: Impact of Jaw-Sucking Movements on Postural Muscles Tension in Young Adults
Source: J Clin Med. 2026 Feb 13;15(4):1464. doi: 10.3390/jcm15041464 (PMC12941828; doi:10.3390/jcm15041464)
Supplement: Supplementary file 1 [file jcm-15-01464-s001.zip › jcm-4008908-supplementary.pdf]

In order to determine the effect of position on the tension of the tested muscles, Friedman's ANOVA was used, and when necessary, the Dunn Bonferroni-Holm post hoc test was used. The effect measure for Friedman's ANOVA was Kendall's W coefficient.

To determine the effect of the test position on the tension of the observed muscle groups, values obtained in the standing, all-fours, and prone and lateral positions were compared, with and without simulated sucking. During the forced sucking condition, no statistically significant differences were observed in the tension levels of the suprahyoid and trapezius muscles with changes in body position. However, significantly lower tension was observed in the gluteus maximus recorded in the lateral position compared to the standing, all-fours, and prone positions. During the standing sucking position, significantly higher tension was also observed in the triceps calf muscle (Table S1). Measurements taken without forced sucking showed significantly lower tension in the suprahyoid and highest tension in the triceps calf muscle in the standing position compared to the other positions. Significantly higher tension was also observed in the gluteus medius in the all-fours position compared to the results of the measurements in the supine position (Table S2).

Table S1. Average and maximum muscle tension during sucking simulation depending on body position, normalized to the standing position

measurement during sucking		Friedman's ANOVA	Kandal's W	Post-Hoc Dunn Bonferroni-Holm p					
				1 vs 2	1 vs 3	1 vs 4	2 vs 3	2 vs 4	3 vs 4
	tension	p		p	p	p	p	p	p
m. suprahyoid	max (%)	0.4981	0.03						
	mean (%)	0.0645	0.09						
m. trapezius	max (%)	0.5784	0.03						
	mean (%)	0.6529	0.03						
m. gluteus maximus	max (%)	**0.0001***	0.37	0.2752	0.5680	**0.0031***	0.1194	**0.0001***	**0.0172***
	mean (%)	**0.0003***	0.26	0.8677	0.8677	**0.0049***	0.3936	**0.0003***	**0.0475***
m. gastrocnemius	max (%)	**0.0001***	0.48	**0.0239***	**0.0001***	**0.0001***	0.1885	0.0695	0.5501
	mean (%)	**<0.0001***	0.48	**0.0426***	**0.0001***	**0.0001***	0.0530	**0.0408***	0.7261

1-4 measurement positions (1. standing position, 2. all-fours position, 3. lying on front, 4. lying on side), *p<0..05

Tabel S2. Average and maximum muscle tension in measurement without sucking simulation, depending on body position, normalized to the standing position

measurement without sucking		Friedman's ANOVA	Kandal's W	Post-Hoc Dunn Bonferroni-Holm p					
				1 vs 2	1 vs 3	1 vs 4	2 vs 3	2 vs 4	3 vs 4
	tension	p		p	p	p	p	p	p
m. suprahyoid	max (%)	**0,0004***	0.28	**0,0140***	**0,0015***	**0,0011***	1,0000	1,0000	1,0000
	mean (%)	**0,0022***	0.22	**0,0097***	**0,0097***	**0,0097***	1,0000	1,0000	1,0000
m. trapezius	max (%)	0,1417	0.08						
	mean (%)	**0,0308***	0.13	1,0000	1,0000	**0,0304***	1,0000	0,1325	0,1885

m. gluteus maximus	max (%)	**0,0031***	0.18	0,2127	0,5192	0,2570	0,3948	**0,0016***	0,0906
	mean (%)	**0,0027***	0.18	0,2127	0,5655	0,2570	**0,0497***	**0,0016***	0,5655
m. gastrocnemius	max (%)	**<0,0001***	0.61	**<0,0001***	**<0,0001***	**<0,0001***	1,0000	1,0000	1,0000
	mean (%)	**<0,0001***	0.62	**<0,0001***	**<0,0001***	**<0,0001***	0,9466	0,8462	0,9466

1-4 measurement positions (1. standing position, 2. all-fours position, 3. lying on front, 4. lying on side), *p<0.05
